# Decreased metabolism and increased tolerance to extreme environments in *Staphylococcus warneri* during long‐term spaceflight

**DOI:** 10.1002/mbo3.917

**Published:** 2019-08-15

**Authors:** Po Bai, Bin Zhang, Xian Zhao, Diangeng Li, Yi Yu, Xuelin Zhang, Bing Huang, Changting Liu

**Affiliations:** ^1^ Medical School of Chinese PLA Beijing China; ^2^ Department of Respiratory Diseases PLA Rocket Force Characteristic Medical Center Beijing China; ^3^ Medical College Nankai University Tianjin China

**Keywords:** differentially expressed genes, long‐term spaceflight, phosphotransferase system, *Staphylococcus warneri*

## Abstract

Many studies have shown that the space environment can affect bacteria by causing a range of mutations. However, to date, few studies have explored the effects of long‐term spaceflight (>1 month) on bacteria. In this study, a *Staphylococcus warneri* strain that was isolated from the Shenzhou‐10 spacecraft and had experienced a spaceflight (15 days) was carried into space again. After a 64‐day flight, combined phenotypic, genomic, transcriptomic, and proteomic analyses were performed to compare the influence of the two spaceflights on this bacterium. Compared with short‐term spaceflight, long‐term spaceflight increased the biofilm formation ability of *S. warneri* and the cell wall resistance to external environmental stress but reduced the sensitivity to chemical stimulation. Further analysis showed that these changes might be associated with the significantly upregulated gene expression of the phosphotransferase system, which regulates the metabolism of sugars, including glucose, mannose, fructose, and cellobiose. The mutation of *S. warneri* caused by the 15‐day spaceflight was limited at the phenotype and gene level after cultivation on the ground. After 79 days of spaceflight, significant changes in *S. warneri* were observed. The phosphotransferase system of *S. warneri* was upregulated by long‐term space stimulation, which resulted in a series of changes in the cell wall, biofilm, and chemical sensitivity, thus enhancing the resistance and adaptability of the bacterium to the external environment.

## Graphical abstract

Taking *S. warneri* as an example, we found that the mutation of bacteria in long‐term spaceflight mainly focused on the adaptation and tolerance such as environmental adaptation, physical resistance, and cell repair to various pressures and these changes should be more obvious or even become stable mutations. But the clinical changes, for example, drug resistance and virulence should not be significant because there is no threat of antibiotics.

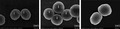

## 1 INTRODUCTION

*Staphylococcus warneri*（*S. warneri*） is a member of coagulase‐negative staphylococci (CNS). Generally, CNS are often considered as simple commensal bacteria due to their rarity in clinical pathology and the absence of virulence factors (Argemi, Hansmann, Prola, & Prévost, 2019), but they are opportunistic pathogens that cause bacteremia and septicemia in immunocompromised people. CNS is the most frequent etiology of bloodstream infection, especially with regard to catheter‐related infections (Hebeisen, Atkinson, Marschall, & Buetti, 2019) and skin and soft tissue infections (Natsis & Cohen, 2018). For example, *S. warneri* can cause arterial embolism in infective endocarditis （Arslan, Saltoglu, Mete, & Mert, 2011）.

The space environment is associated with a variety of unique stressors, such as low gravity, strong radiation such as ultraviolet radiation and heavy‐ion radiation, and a weak magnetic field, which can accelerate the microbial mutation rate and lead to many different phenotypes (Su, Chang, & Liu, 2013). Some microbial isolates were collected from cosmonauts and filter debris during orbital spaceflights on the International Space Station (Checinska et al., 2015), and staphylococci were the most frequently isolated bacteria from the Mir space station (Novikova, 2004). In addition, many studies have shown that the extreme environment of space plays an important role in dysregulation of the human immune system (Crucian et al., 2018). During spaceflight, staphylococci are carried into the extraterrestrial environment by the space crew. In the stressful space environment, these bacteria may develop unexpected traits that could have important effects on the spaceflight mission and pose potential threats to general public health. A previous study found that the expression of the *rpoB* gene of *S. epidermidis* was upregulated after spaceflight, and its mutation frequency increased considerably (Fajardo‐Cavazos & Nicholson, 2016). These uncertain variations increase the potential risk of pathogenic CNS infection in humans and have become a hot spot in the study of space microbiology. Similar to other CNS using adhesion factors for the colonization of foreign body biomaterials (Becker, Heilmann, & Peters, 2014), *S. warneri* can easily adhere to artificial equipment or to the condensate in water pipelines, which increases the risk of infection for astronauts. The above effects together with the space environment and spacecraft conditions could compromise the balance between the human body and human microbiome, induce immune dysregulation in astronauts, and eventually increase the risk for microbial infections (Cervantes Jorge & Bo‐Young, 2016). Thus, the risk associated with *S. warneri* may be greater in space than on the ground. In addition, to date, there has been no research to distinguish the influence of short and long spaceflights on microorganisms. Compared to a short spaceflight, a long flight increases the exposure of the bacteria to microgravity and space radiation, which may have unknown effects on bacterial phenotypes and gene expression. It is unclear whether the microgravity or elevated radiation doses experienced during a long spaceflight can increase the mutation rate of *S. warneri* and whether this mutation is stable. In general, one would expect that these bacteria may reduce their metabolic activities to adapt and survive in the severe space environment during a long flight. However, all of these questions remain unanswered. In this study, an *S. warneri* isolate was carried by the Shenzhou‐10, 11 spacecraft, and Tiangong‐2 space laboratory. This study provided us with a unique opportunity to explore the underlying mechanism of bacterial tolerance in extreme environments. Accordingly, the phenotypic, genomic, transcriptomic, and proteomic analyses were combined to examine the influence of the long‐term spaceflight on this bacterium.

## 2 METHODS

### 2.1 Bacterial strains and culture conditions

The ancestral *S. warneri* strain (designated SWO) was isolated from condensate water obtained from the Shenzhou‐10 spacecraft (which was launched on 11 June 2013 and had a 15‐day spaceflight) and stored at −80℃ with 20% glycerol. SWO was prepared by overnight growth in Luria‐Bertani liquid medium (LB) in Vapour‐bathing Constant Temperature Vibrator (Ke Xi, Chang Zhou, China). The LB medium contained tryptone (10 g/L), yeast extract (5 g/L), NaCl (10 g/L), and agar powder (15 g/L), and the pH of the medium was adjusted to 7.0–7.2 (Guo et al., 2014). After cultivated under the conditions of 37°C and 200 rpm/min overnight, the cultures were diluted in LB to a working concentration of 10^8^ CFU/ml and put in two special plastic containers designed for this study as previously described (Su et al., 2014) prior to use. The strain (designated SWS) in one plastic container was stored in a liquid nitrogen transport tank and left the laboratory of the Chinese PLA General Hospital at 08:25 on 15 September 2016 (GMT + 8). The strain was transported to Jiuquan Satellite Launch Center (JSLC) by military plane and was finally carried into the space environment by the incubator of Tiangong‐2 space laboratory launched at 22:40 on 15 September 2016 (GMT + 8). After 64 days of flight at an approximate apogee distance of 393 km, SWS was returned to Earth by the Shenzhou‐11 spacecraft (which was launched on 17 October 2016 and docked with the Tiangong‐2 space laboratory on 19 October). The return capsule of the Shenzhou‐11 spacecraft completed the experimental task and left the Tiangong‐2 space laboratory at 12:41 on 17 November and landed at Siziwang Banner at 14:07 on 18 November (GMT + 8). The microbiological sample was quickly removed into a liquid nitrogen transport tank and transported to Beijing by military plane. The sample arrived at the laboratory of the Chinese PLA General Hospital at 20:02 on 18 November (GMT + 8). The strain (designated SWG) in the other plastic container was cultured on the ground as a control experiment. This plastic container was simultaneously put into an incubator to simulate the environment of the space laboratory, The parameters of the two incubators are as follows: temperature of 37°C, humidity 43%, and oxygen content 21%. The incubators of land are adjusted in accordance with the data reported each hour sent back by the spacelab's monitoring device, so except space environment, all other culture conditions were identical between SWG and SWS groups. The three strains were revived immediately after the arrival of SWS at the laboratory, and a series of assays were performed to explore the effect of the space environment on *S. warneri*, including analyses of morphology, biophysical features, and growth rate and genomic, transcriptomic and proteomic assays. For transcriptomic and proteomic analyses, we sequenced the SWG and SWS strains three times each.

### 2.2 Phenotypic characteristics

#### 2.2.1 Scanning electron microscopy

The *S. warneri* strains, grown in LB medium, were washed three times with PBS (pH 7.4) and fixed with 4% glutaraldehyde overnight. After washing three times again with PBS (pH 7.4), the three samples were dehydrated with an increasing gradient of ethanol and critical point dried. After coating with gold–palladium, the specimens were examined with an FEI Quanta 200 *SEM* scanning electron microscope (FEI).

#### 2.2.2 Drug susceptibility testing

Drug susceptibility testing (DST) was performed using the disk diffusion method. In brief, based on the CLSI M100‐S24 document (CLSI, 2014), 12 antibiotics were selected to test the resistance of the three *S. warneri* strains, namely ertapenem (ETP), aztreonam (ATM), ciprofloxacin (CIP), linezolid (LZD), tobramycin (TOB), trimethoprim–sulfamethoxazole (SXT), amikacin (AK), cefazolin (KZ), cefepime (FEP), vancomycin (VA), ceftriaxone (CRO), and ampicillin/sulbactam (SAM). The entire surface of the Petri dish containing LB was covered with the required inoculum (10^7^–10^8^ CFU/ml), and the plate was dried for 15 min before the disks were placed on the surface. Following incubation for 18 hr at 37℃, the zone diameters were measured according to the standard protocol. All experiments were performed in technical triplicate.

#### 2.2.3 Carbon source utilization and chemical sensitivity assay

We performed carbon source utilization and chemical sensitivity tests using the 96‐well Biolog GENIII MicroPlate, which included 71 carbon sources and 23 chemical compounds. Briefly, the bacterial culture was picked up from the surface of the BUG1B agar plate (Biolog) using a sterile cotton‐tipped swab and inoculated into IF‐A Inoculum (Biolog). The target cell density of the inoculum was set to 90%–98% by a turbidimeter (BioMe'rieux). Then, 100 ml of the inoculum was added into each well of the 96‐well GEN III MicroPlate (Biolog). After incubating for 24 hr at 37°C, the OD_630_ of each well was automatically measured with a Biolog microplate reader. All experiments were performed in technical triplicate.

#### 2.2.4 Growth rate

Bacterial growth was monitored using the Bioscreen C system and BioLink software (Lab Systems). The *S. warneri* strains were grown overnight in LB liquid medium at 37°C A 20‐µl aliquot of an overnight culture with a concentration of 10^6^ CFU/ml was cultivated in Bioscreen C (Lab Systems) in 200‐well microtiter plates (honeycomb plates). The plates were continuously shaken at the maximum amplitude and incubated for 24 hr at 37℃ with 350 µl of fresh LB growth medium per well. The OD_630_ was measured every 2 hr. A blank well with 370 µl of LB was also included. All experiments were performed in technical triplicate.

#### 2.2.5 Biofilm assay

One microliter of culture was added to 99 µl of LB liquid medium and cultured overnight. The following day, 100 ml of the overnight culture was inoculated in 5 ml of LB medium and shaken at 37°C for 3 hr until the OD_600_ reached 0.5. Then, 100 µl of this culture was added into a well of a 96‐well microtiter dish made of PVC (Falcon 3,911) and incubated at 30°C for 30 min. Nonattached bacteria were removed by discarding the media/cells, and the wells were rinsed 3–4 times by immersing the plate in a tub of water and discarding the water from the wells. The plate was patted dry on paper towels to remove the excess dye. Then, 120 ml of a 0.1% solution of crystal violet was added, and the plate was incubated at 30°C for 30 min. The wells were rinsed 3–4 times as described above. Then, 200 ml of 95% ethanol was added, and an attempt was made to solubilize all the CV adhered to the walls of the wells. Then, 125 ml was transferred from each well to a fresh polystyrene microtiter dish (Costar), and the OD_560_ was determined in a microplate reader. All experiments were performed in technical triplicate.

### 2.3 Genome sequencing

#### 2.3.1 Genome sequencing and assembly

The SWO DNA samples were obtained using the conventional phenol–chloroform extraction method (Javadi et al., 2014). After electrophoretic detection, qualified DNA samples were sheared into small fragments by Covaris S/E210, and then, the overhangs of the DNA fragments were converted into blunt ends using T4 DNA polymerase, Klenow fragment, and T4 polynucleotide kinase. An “A” base was added onto the 3’ end of the blunt phosphorylated DNA fragments, and adapters were ligated to the ends of the fragments. After gel electrophoresis, the purified fragments were enriched and amplified by polymerase chain reaction (PCR). Finally, a 10‐kb SMRTbell library was constructed. The genome was sequenced using a PacBio RS II platform and an Illumina HiSeq 4,000 platform at the Beijing Genomics Institute (BGI, Shenzhen, China). Four SMRT Cell zero‐mode waveguide arrays for sequencing were used by the PacBio platform to generate the subread set. PacBio subreads with lengths <1 kb were removed. The program Pbdagcon was used for self‐correction. Draft genomic unities, which were uncontested groups of fragments, were assembled using the Celera Assembler against a high‐quality corrected circular consensus sequence subread set. To improve the accuracy of the genome sequences, the GATK and SOAP tool packages (SOAP2, SOAPsnp, and SOAPindel) were used to make single‐base corrections. To search for the presence of any plasmid, the filtered Illumina reads were mapped to the bacterial plasmid database using SOAP.

#### 2.3.2 Genome component prediction

Gene prediction was performed on the SWO genome assembly by Glimmer3 with hidden Markov models. tRNA, rRNA, and sRNA identification was performed by using tRNAscan‐SE, RNAmmer, and the Rfam database, respectively. The tandem repeat annotation was obtained using the Tandem Repeat Finder, and the minisatellite DNA and microsatellite DNA were selected based on the number and length of repeat units. The Genomic Island Suite of Tools (GIST) was used for genomic island analysis with the IslandPath‐DIOMB, SIGI‐HMM, and IslandPicker methods. Prophage regions were predicted using the PHAge Search Tools (PHAST) web server, and CRISPR identification was performed using CRISPRFinder.

#### 2.3.3 Gene annotation and protein classification

The best hit was obtained using the BLAST alignment tool for function annotation. Seven databases, namely Kyoto Encyclopedia of Genes and Genomes (KEGG), Clusters of Orthologous Groups (COG), Non‐Redundant Protein database (NR), Swiss‐Prot (Ashburner et al., 2000), Gene Ontology (GO), TrEMBL, and EggNOG, were used for general function annotation. For pathogenicity and drug resistance analyses, virulence factors and resistance genes were identified based on the core dataset in the Virulence Factors of Bacterial Pathogens database (VFDB), Antibiotic Resistance Genes database (ARDB), Pathogen‐Host Interactions (PHI)‐base database, and Carbohydrate‐Active Enzymes database (CAED).

### 2.4 Comparative genomic analysis

#### 2.4.1 SNP detection

Using the alignment software MUMmer (Kurtz et al., 2004), each query sequence from SWS and SWG was aligned with the reference sequence. The variation sites between the query sequence and reference sequence were identified and filtered preliminarily to identify potential SNP sites. Sequences with lengths of 100 bp on both sides of the SNP in the reference sequence were extracted and aligned with the assembly results to verify SNP sites using BLAST (de Leeuw et al., 2012). If the length of the aligned sequence was shorter than 101 bp, the SNP was considered noncredible and was removed; if the extracted sequence was aligned with the assembly results several times, the SNP was considered to be located in the repeat region and was removed. BLAST, TRF, and Repeatmask software were used to predict SNPs in repeat regions. Credible SNPs were identified by filtering SNPs located in repeat regions.

#### 2.4.2 InDel detection

With LASTZ (Chiaromonte, Yap, & Miller, 2002; Harris, 2007) software, the reference and query sequence from CAED were aligned. After a series of treatments with axt‐correction, axt‐Sort, and axt‐Best, the best alignment results were chosen, and the InDels were preliminarily identified. One hundred and fifty base pairs upstream and downstream of the InDel site in the reference sequence were extracted and then aligned with the query reads. The alignment result was verified with BWA (Li et al., 2009) and SAMtools.

### 2.5 Transcriptome sequencing and comparison

#### 2.5.1 Sequencing and filtering

Bacterial cells were collected after centrifugation at 8,000 *g* for 5 min at 4°C and mixed with TRIzol reagent thoroughly. The samples were centrifuged at 10,000 *g* for 5 min at 4°C. The supernatant was added to chloroform, shaken for 15 s, and centrifuged at 10,000 *g* for 10 min at 4°C. Then, the supernatant was transferred into a tube containing isopropanol and centrifuged at 13,600 *g* for 20 min at 4°C. After removal of the supernatant, the sample was mixed with ethanol and centrifuged at 12,000 *g* for 3 min at 4°C. Subsequently, the supernatant was removed, and the sample was centrifuged at 12,000 *g* for 20 s at 4°C. After removal of the residual liquid and air‐drying, the RNA pellet was dissolved in RNase‐free water. Total RNA from SWS and SWG was extracted using the TIANGEN RNAprep Pure Kit (Agilent 2100 Bioanalyzer) following the manufacturer's instructions. The mRNAs containing poly‐A were purified using poly‐T oligo‐attached magnetic beads. The purified mRNAs were fragmented into small pieces (~200 bp) using divalent cations. The RNA fragments were used to generate first‐strand cDNA by reverse transcriptase and random primers. Then, the second‐strand cDNA was created using DNA Polymerase I and RNase H. The cDNA fragments were enriched with PCR amplification and quantified by Qubit 2.0. Finally, the cDNA libraries were constructed. The libraries were sequenced using BGISEQ‐500. Raw reads were filtered using the software SOAPnuke (version 1.5.2), and clean reads were acquired by removing adapter reads, poly‐N reads, and reads with 40 bp of low quality (<Q 15) base numbers.

#### 2.5.2 Gene expression statistics

We mapped clean reads to the reference strain using HISAT (version 2.0.4) and Bowtie2 (version 2.2.5) (Li & Dewey, 2011) and then calculated the gene expression level with RSEM (Langmead & Salzberg, 2012). Cluster analysis of gene expression was performed using Cluster software (de Hoon, Imoto, Nolan, & Miyano, 2004) (version 3.0), and the results of cluster analysis were visualized with Java TreeView (Saldanha, 2004).

#### 2.5.3 Differential gene expression analysis

Differential gene expression was analyzed using the DESeq method based on the Poisson distribution and performed as described by Wang (Wang, Feng, Wang, Wang, & Zhang, 2010). *p*‐values were corrected to *Q*‐values as previously described (Storey & Tibshirani, 2003) to improve the accuracy of identification of differentially expressed genes (DEGs). Genes with fold changes ≥2 and *Q*‐values ≤0.001 in two different samples were defined as DEGs, and then, the identified DEGs were enriched and clustered according to the GO and KEGG databases.

### 2.6 Proteomic analysis

#### 2.6.1 Polypeptide preparation

The samples in liquid LB medium were collected by centrifugation at 25,000× *g* for 15 min at 4°C. After the supernatant was discarded, the samples were thoroughly mixed with 1 ml of PBS and centrifuged at 25,000× *g* for 15 min at 4°C. The supernatant was removed, and lysis buffer (8 M Urea, 40 mM Tris–HCl or TEAB, pH 8.5) was added to the samples. The suspension was sonicated for 1 min and centrifuged at 25,000× *g* for 15 min at 4°C. After 10 mM DDT was added, the supernatant was incubated for 1 hr at 56°C. Subsequently, the samples were incubated with 55 mM IAM for 45 min in the dark room for alkylation. Finally, the supernatant containing proteins was extracted after centrifugation at 25,000× *g* for 15 min at 4°C. To perform quantitative proteomics and examine differences in the protein profiles of these samples, we used high‐throughput technology based on iTRAQ combined with two‐dimensional liquid chromatography–tandem mass spectrometry (2D‐LC‐MS/MS), which was performed as previously described (Unwin, Griffiths, & Whetton, 2010). After extraction, quantification, SDS–polyacrylamide gel electrophoresis, and trypsin digestion, the protein samples were processed into polypeptides and labeled with iTRAQ reagents. The polypeptides were separated by liquid phase separation and freeze‐dried by a liquid phase system (SHIMADZU LC‐20AB) and then separated by nanoliter liquid chromatography (SHIMADZU LC‐20AD). After liquid phase separation, the peptides were ionized by nanoESI (The parameters for MS analysis are listed as following: electrospray voltage: 1.6 kV; precursor scan range: 350–1,600 m/z at a resolution of 70,000 in Orbitrap; MS/MS fragment scan range: >100 m/z at a resolution of 17,500 in HCD mode; normalized collision energy setting: 27%; dynamic Exclusion time: 15 s; Automatic gain control (AGC) for full MS target and MS2 target: 3e6 and 1e5, respectively; the number of MS/MS scans following one MS scan: 20 most abundant precursor ions above a threshold ion count of 20,000) and injected into a Q‐Exactive mass spectrometer (Thermo Fisher Scientific) for DDA (data‐dependent acquisition)‐mode detection to finally obtain raw data.

#### 2.6.2 iTRAQ quantification

The raw data were compared with the corresponding database by Mascot software for protein identification. To determine whether the data were qualified, credible protein identification results were obtained. To quantify the iTRAQ data, we used IQuant (Wen et al., 2014) software, which integrated the Mascot Percolator (Brosch, Yu, Hubbard, & Choudhary, 2009) algorithm, to improve the identification rate. To assess the confidence of peptides, the PSMs were pre‐filtered at a PSM‐level FDR of 1%. Then based on the parsimony principle (explain all identified peptides with minimal protein), identified peptide sequences are assembled into a set of confident proteins. In order to control the rate of false‐positive at protein level, an protein FDR at 1%, which is based on Picked protein FDR strategy (Savitski, Wilhelm, Hahne, Kuster, & Bantscheff, 2015), will also be estimated after protein inference (Protein‐level FDR <= 0.01). In conclusion, we used iTRAQ quantitative analysis to screen out significantly different proteins of interest from the results. Finally, GO functional annotations and KEGG pathway enrichment analysis of differentially expressed proteins (DEPs) were carried out.

## 3 RESULTS

### 3.1 Phenotypic characteristics

#### 3.1.1 Morphology

**Figure 1:**
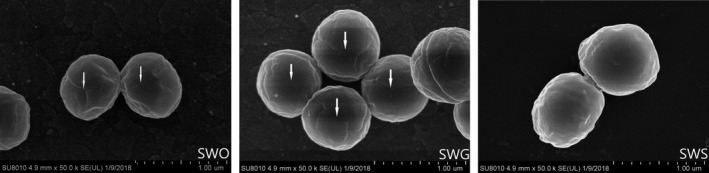
*SEM* analysis of strain morphology. Distinct cracks (white arrows) were observed on the surfaces of SWO and SWG, while no cracks were observed on the surface of SWS. (scale bars :4.9mm × 50.0 k)

The three strains showed no significant difference under the optical microscope, but when scanning electron microscopy (*SEM*) was used to monitor single‐cell morphology, distinct cracks were observed on the cell surfaces of SWO and SWG but not SWS (Figure 1).

#### 3.1.2 Antibiotic susceptibility test

The results of the disk diffusion assays revealed that the three strains were sensitive to all the tested antibiotic disks, and no obvious differences were observed among the strains (Table 1).

**Table 1:** Antibiotic susceptibility analysis of the three strains

Antibiotic	Inhibition zone diameter (mm)		
	SWO	SWG	SWS
Ertapenem	30/S	30.5/S	29.8/S
Aztreonam	0/R	0/R	0/R
Ciprofloxacin	30.4/S	29.5/S	30.5/S
Linezolid	32/S	30/S	31.6/S
Tobramycin	15.3/S	15/S	15.5/S
Trimethoprim–sulfamethoxazole	30.5/S	29.5/S	30.5/S
Amikacin	24/S	23.5/S	22.8/S
Cefazolin	26.5/S	25.5/S	25.9/S
Cefepime	27/S	26.6/S	26.5/S
Vancomycin	17.8/S	17/S	17.5/S
Ceftriaxone	26.8/S	26.5/S	25.9/S
Ampicillin/sulbactam	20.5/S	20/S	20.3/S

#### 3.1.3 Chemical sensitivity and carbon source utilization assays

**Figure 2:**
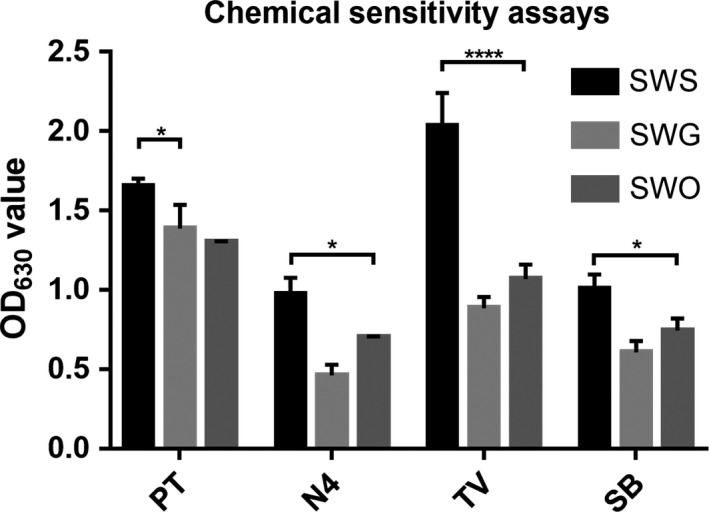
Chemical sensitivity assays of the three strains. The SWO, SWG, and SWS strains were incubated in a Biolog GENIII MicroPlate at 37°C for 24 hr with LB medium and then tested for carbon source utilization and chemical sensitivity. X‐axis: potassium tellurite (PT), niaproof4 (N4), tetrazolium violet (TV), and sodium butyrate (SB)

The chemical sensitivity assay showed that SWS exhibited increased tolerance to potassium tellurite (PT) (SWS vs. SWG, *p = *0.0421), niaproof 4 (N4) (SWS vs. SWO, *p* = 0.0436), tetrazolium violet (TV) (SWS vs. SWO, *p* < 0.0001), and sodium butyrate (SB) (SWS vs. SWO, *p* = 0.0482) compared to SWG and SWO (Figure 2). However, there were no significant differences between SWO and SWG. Carbon source utilization assays showed that there was no significant difference among the three strains.

#### 3.1.4 Growth rate assay

**Figure 3:**
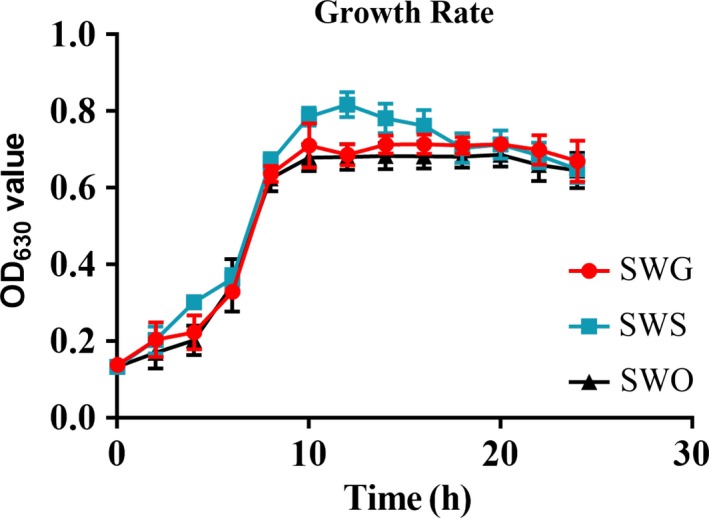
Growth rates of the three strains in LB medium. The SWO, SWG, and SWS strains were cultured in 20 ml of LB medium at 37°C for 24 hr with agitation. The OD_630_ was measured every 2 hr for 24 hr

A slight difference was observed among the three strains in terms of growth rate and maximum OD_630_ values, but the difference was not significant (Figure 3).

#### 3.1.5 Biofilm assay

**Figure 4:**
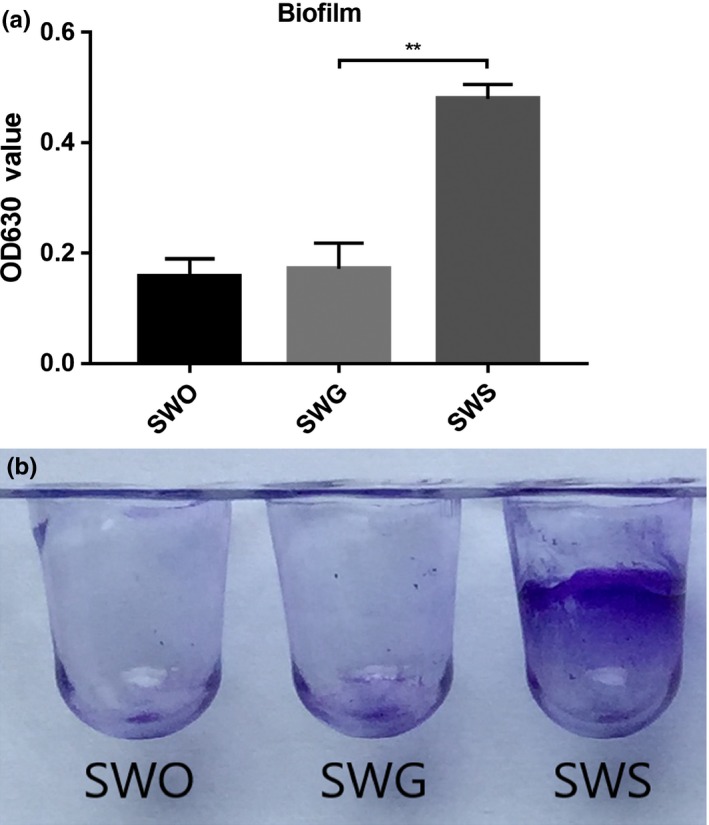
Analysis of biofilm formation ability via crystal violet staining. The three strains were cultured in 96‐well polystyrene microtiter plates at 37°C for 24 hr. Biofilm formation ability was measured by determining the OD_560_ of crystal violet

The biofilm formation ability of bacteria is considered to be a significant phenotype because it is associated with host colonization, antibiotic resistance, and environmental persistence (Andrade et al., 2014; Lee et al., 2014). Therefore, we performed a biofilm test to estimate the biofilm formation ability of the three *S. warneri* strains. SWS showed the strongest biofilm formation ability, and SWG and SWO exhibiting hardly any biofilm formation (*p* = 0.0013) (Figure 4).

### 3.2 Genome sequencing, assembly, and annotation

We performed complete genetic prediction and identification for SWO. After the complete genome of SWO was assembled, the results were used as a reference for comparative genomic analysis of SWS and SWG. Using Glimmer software (Delcher, Bratke, Powers, & Salzberg, 2007), we identified 2,486 genes with a total length of 2,183,598 bp, which constituted 84.88% of the genome. The accession number of SWO is CP033098‐CP033101. In addition, 5,745 bp of minisatellite DNA and 213 bp of microsatellite DNA sequences were identified, constituting 22.33% and 0.83% of the genome, respectively.

**Figure 5:**
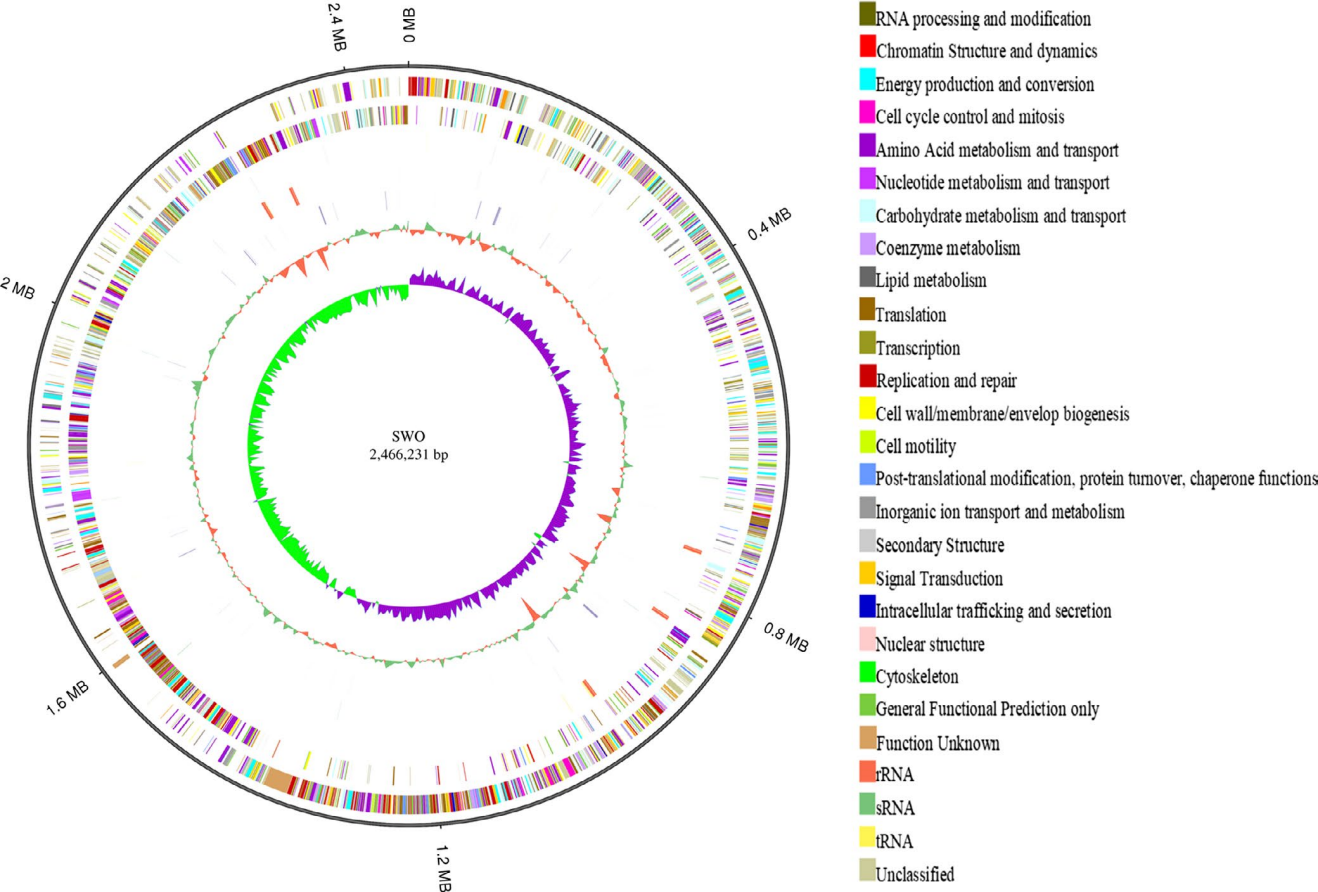
Genome map of SWO (ncRNA, COG annotation, GC content, and GC skew). From outer to inner, the 1st circle shows the genome size; the 2nd circle shows the COG function of the forward strand gene, and each color represents a function classification; the 3rd circle shows the function of the reverse strand gene; the 4th circle shows the ncRNA results of the forward strand containing tRNA, rRNA, and sRNA; the 5th circle shows the ncRNA results of the reverse strand; the 6th circle shows repeat sequences; the 7th circle shows the GC content; and the 8th circle shows the GC skew ((G‐C)/(G + C), green > 0, purple < 0)

All genes were annotated against popular functional databases, including 65.32% of the genes in the GO database (Kanehisa, Goto, Furumichi, Tanabe, & Hirakawa, 2010), 75.98% of the genes in the COG database (Ashburner et al., 2000), 61.7% of the genes in KEGG (Tatusov, Galperin, & Natale, 2000), 15.08% of the genes in the NOG database, and 99.07% of the genes in the NR database. Moreover, 16 genes were identified in the CAED (Cantarel et al., 2009), 188 genes in the PHI‐base (Pathogen‐Host Interaction) database (Winnenburg et al., 2006), 15 genes in the ARDB (Antibiotic Resistance Genes database), and 122 genes in VFDB (Virulence Factors database) (Chen et al., 2005). The genome map of SWO is shown in Figure 5.

### 3.3 Comparative genomic analysis

SWO was used as the reference strain to detect variations (SNPs and InDels) in SWS and SWG by using comparative genomic sequencing (Table 2). The accession number for the resequencing data of SWS is SAMN10255186, and that for SWG is SAMN10250545. Subsequently, we conducted a structural variation analysis of SWS and SWG compared to SWO.

**Table 2:** Statistics of comparative genome sequencing

Sample	SWS	SWG
Raw read statistics		
Total reads	2,685,888	2,776,954
Sequencing depth (X)	132	133
Genome coverage (%)	100%	100%
Assembly statistics		
Chromosome size	2,524,548	2,518,925
No. of scaffolds	22	17
Largest scaffold length	687,388	1,265,212
N50 scaffold length	318,178	1,265,212
G + C content (%)	32.50%	32.50%

The raw variation sites were identified and then filtered with strict standards to detect potential InDels. After a series of filtering conditions, we identified 15 InDels between SWS and SWG, including nine InDels in intergenic regions and six in a coding region. Four InDels were unique to SWS; five were unique to SWG; and all nine were located in the intergenic regions. The CDS InDel was identified in SWOGL000595, SWOGL002370, and SWOGL002369, which are annotated as domain‐containing proteins.

### 3.4 Transcriptomic analysis

**Figure 6:**
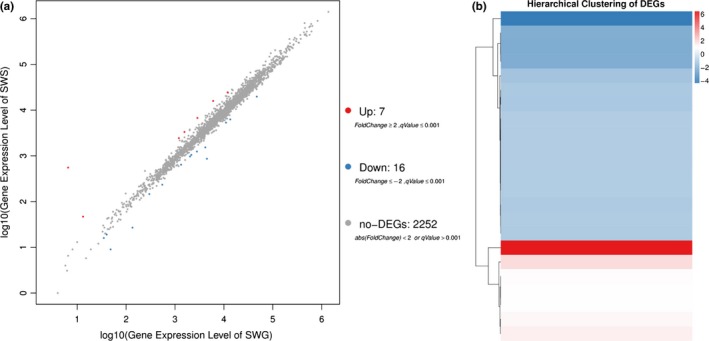
Differential transcriptomic expression. (a) These diagrams represent the number of genes that are specifically expressed in SWS and SWG. (b) Hierarchical clustering of DEGs. The X‐axis represents the difference comparison for cluster analysis, and the Y‐axis represents the differentially expressed gene. Colors represent the difference multiples converted by log2

The accession numbers for the transcriptomic data of SWS and SWG are SAMN10255187 and SAMN10255193, respectively. The average output for SWS and SWG was 21.87 and 21.86 M. A total of 2,271 and 2,274 genes were expressed in SWS and SWG, including 2,270 genes identified in both strains. Both upregulated and downregulated genes were identified. In SWS, approximately seven genes were upregulated, and 16 genes were downregulated (Figure 6). Simultaneously, we found that downregulated genes significantly outnumbered upregulated genes, suggesting that gene expression and metabolism were inhibited in SWS.

We observed that there were not many changes in gene expression between the two strains, suggesting that *S. warneri* was relatively stable under the stimulation of the external environment. In other words, this strain exhibited relatively strong resistance to the external environment.

Differentially expressed genes were enriched and clustered according to GO and KEGG analyses. For GO analysis, the transcriptome of SWS was characterized by regulation of four statistically significant DEGs involved in transporter activity, a subfunction of molecular function. The upregulated and downregulated genes were summed and compared with unchanged genes. We found that this function was significantly downregulated by three genes and upregulated by 1 gene (*p* = 0.0435) (Table 3).

**Table 3:** GO functional annotation and regulation of four DEGs

Gene ID	Regulation	Molecular function
SWOGL000019	Down	Transporter activity
SWOGL000052	Down	Protein‐*N*(PI)‐phosphohistidine‐sugar phosphotransferase activity
SWOGL001384	Down	Methanol sensitive ion channel activity
SWOGL001954	Up	Protein‐*N*(PI)‐phosphohistidine‐fructose phosphotransferase system transporter activity Sugar:proton symporter activity

Using the KEGG orthology‐based annotation system to identify metabolic pathways, we identified two statistically significant DEGs (*p* = 0.0027) (SWOGL001955, SWOGL001954) in fructose and mannose metabolism (Table 4). The gene SWOGL001954 was also enriched in the other three KEGG categories. In contrast to the GO results, the KEGG pathways were all upregulated.

**Table 4:** KEGG functional annotation and regulation of four DEGs

Gene ID	Regulation	Fructose and mannose metabolism
SWOGL001955	Up	1‐phosphofructokinase
SWOGL001954	Up	PTS system, fructose‐specific IIA component
		PTS system, fructose‐specific IIB component
		PTS system, fructose‐specific IIC component

### 3.5 Comparative proteomic analysis

**Figure 7:**
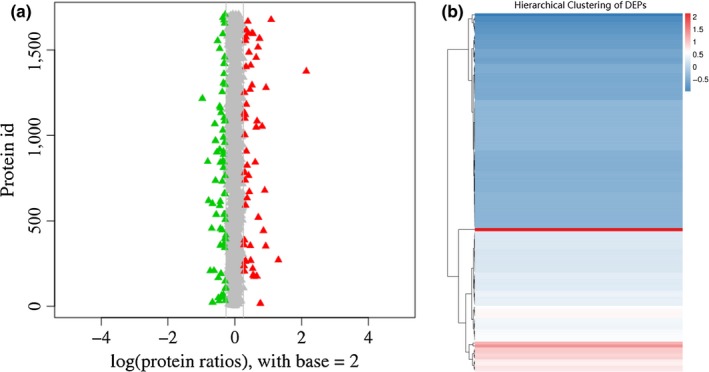
Differential protein expression. (a) Protein ratio distribution of SWS and SWG. (b) Hierarchical clustering of DEPs

There were totally 1,771 proteins were identified and quantified. Relatively quantitative analysis identified 120 DEPs, including 72 downregulated and 48 upregulated proteins (Figure 7).

**Figure 8:**
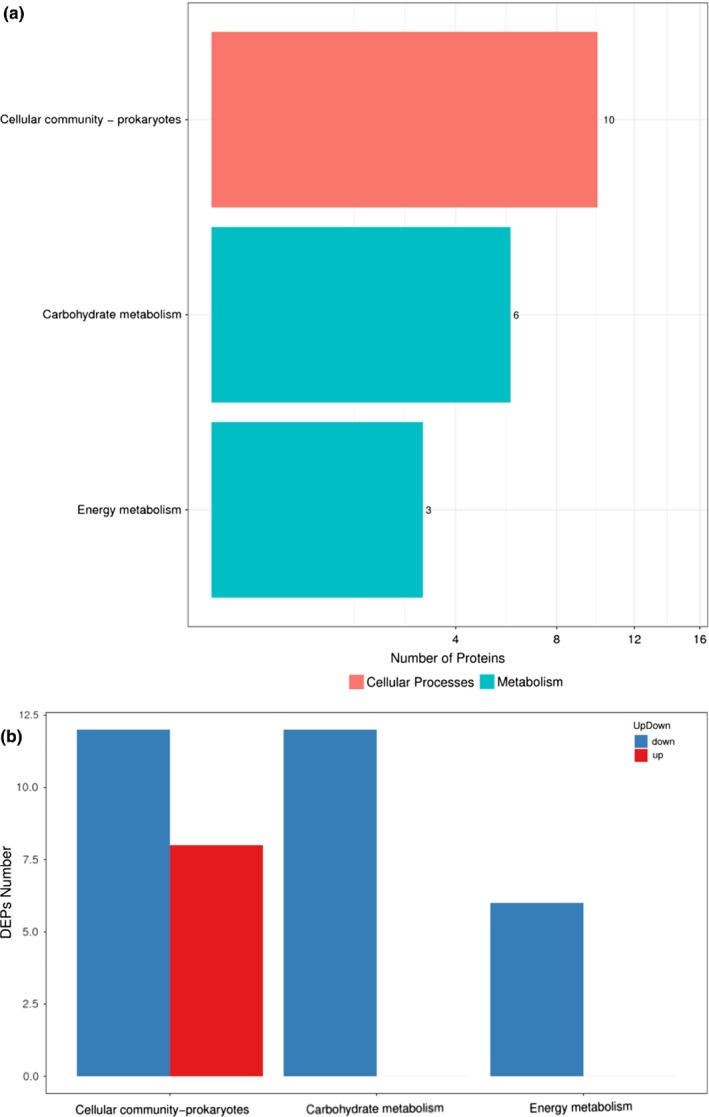
Differential proteomic analysis. (a) Clustered DEPs in KEGG pathways of SWS. The X‐axis represents the number of proteins corresponding to the GO functions. The Y‐axis represents KEGG pathway functions. (b) The regulation conditions of DEPs enriched in KEGG pathways of SWS. The X‐axis represents the number of proteins corresponding to KEGG pathway functions. The Y‐axis represents KEGG pathway functions

Subsequently, DEPs were enriched and clustered according to GO and KEGG analyses. Regarding the GO function category, it was clear that the expression of proteins involved in functions such as membrane (*p* = 0.0039), integral component of membrane (*p* = 0.0016), intrinsic component of membrane (*p* = 0.0017), cell periphery (*p* = 0.0023), and membrane part (*p* = 0.0024) was statistically significant. The upregulated and downregulated proteins were summed and compared with unchanged proteins. The downregulation of DEPs in GO categories was significant, and almost all the proteins enriched in biological process and molecular function were downregulated. With respect to KEGG functions, a significant difference was observed in the following pathways: cellular community–prokaryotes (*p* < 0.0001), carbohydrate metabolism (*p* = 0.0002), and energy metabolism (*p* = 0.0321) (Figure 8a). Unlike the transcriptomes, the KEGG pathways of the DEPs were downregulated (Figure 8b), including carbohydrate metabolism and energy metabolism.

## 4 DISCUSSION

The effect of the space environment on microorganisms has been studied extensively, but there are few reports on the effect on *Staphylococcus* (Fajardo‐Cavazos & Nicholson, 2016; Guo et al., 2014). In addition, the effect of long‐term flight on bacteria has not been studied. A long‐term spaceflight increases the risk of gene mutation of bacteria by extended exposure to microgravity and strong high‐energy radiation. In this study, we took advantage of this rare opportunity to study these two issues. *S. warneri* was carried as normal bacterial flora on the skin mucous membrane of an astronaut from Shenzhou‐10. SWS had experienced two flights, one for 15 days and another for 64 days (see the description in the Methods for details). Some limited but significant changes between SWS/SWG/SWO were observed at the genetic level, which led us to the following two conclusions.

1. Changes caused by the space environment occur mostly in genes associated with resistance and adaptation to external stimulation. This effect can be demonstrated by the changes in the phenotypes of SWS, including cell walls, biofilms, and chemical sensitivity. The main component of the staphylococcal cell wall is peptidoglycan, and the mechanical strength of the cell wall of *S. warneri* is dependent on the presence of peptidoglycan. Thus, the cell wall of *S. warneri* is closely associated with the metabolic pathway of sugars. Biofilms, which are an important feature of *Staphylococcus* species, are defined as sessile microbial communities in which cells are attached to a surface or to other cells and embedded in a protective extracellular polymeric matrix. This mode of growth exhibits altered physiologies with respect to gene expression and protein production (Chatterjee, Chatterjee, Dey, Kundu, & Dey, 2014; Kiedrowski & Horswill, 2011; Parsek & Singh, 2003). A major constituent of the biofilm matrix is polysaccharide intercellular adhesin (PIA), and the formation of PIA depends on the phosphotransferase system (PTS), a distinct method used by bacteria for sugar uptake. In this system, the source of energy is phosphoenolpyruvate (PEP), which is involved in the transport of many sugars into bacteria, including glucose, mannose, fructose, and cellobiose. Although there is a general downregulation trend in gene expression and protein products in SWS, there remains significant upregulation of the PTS in glucose metabolism. There are four genes involved in glucose metabolism, among which three were upregulated and one was downregulated. Upregulation of the SWOGL001954 gene plays an important role in the expression of the PTS. According to GO and KEGG database annotation, SWOGL001954 participates in not only the composition of the fructose‐specific IIA/IIB/IIC components but also the transport of these complexes in the PTS. This finding indicates that the functions of SWS in the manufacture, utilization, and transport of glucose metabolism associated with the PTS were significantly enhanced. Therefore, it is believed that changes in the biofilm and cell wall are directly associated with PTS enhancement in SWS. Peng et al. (2017 found that the PTS in *Enterococcus faecalis* helped increase the resistance of this species to environmental stress. Therefore, it is reasonable to think that the decreased sensitivity of SWS to chemical stimulation is also associated with the enhancement of PTS expression. Chemical resistance might be achieved via a series of changes in biofilms and cell walls. DEPs enriched in KEGG showed a decrease in glucose metabolism, and we found the only DEP correlated with PTS to be lactose/cellobiose transporter subunit IIA (protein ID: WP_002450492.1). This protein is an enzyme with regulatory function. For example, at low glucose concentrations, the enzyme accumulates and activates membrane‐bound adenylate cyclase. When the glucose concentration is high, the enzyme is mostly dephosphorylated and inhibits adenylate cyclase, glycerol kinase, lactose permease, and maltose permease. Therefore, this protein is a bidirectional regulator in the PTS. All the other DEPs that were downregulated in the KEGG function glucose metabolism were associated with galactose metabolism, which is independent of the PTS. Galactose can be used for prevention of biofilm formation by targeting the activity of autoinducer 2 (AI‐2), a quorum sensing molecule that is involved in virulence and biofilm formation (Ryu, Sim, Sim, Lee, & Choi, 2016). This change confirms that the thickening of the biofilm may be associated with the downregulation of galactose metabolism.
2. The results of transcriptomic and proteomic analyses indicate that SWS exhibits a relatively low metabolic rate. Therefore, it is reasonable to believe that SWS can increase its resistance to the external environmental stimuli by lowering its own metabolism, and this change has indirectly led to, even after a long spaceflight, the influence of the space environment on staphylococci to remain relatively weak, which is consistent with the results of Guo et al., (2015. In addition, the results of some other studies on the resistance of staphylococci in the space environment were inconsistent. Tixador (Tixador et al., 1985) found that the lowest inhibitory concentrations of benzocillin, erythromycin, and chloramphenicol for *S. aureus* increased. Similar changes were observed in *S. epidermidis*, the most abundant *Staphylococcus* species in the space environment (Venkateswaran et al., 2014)**.** A study by Fajardo‐Cavazos (Fajardo‐Cavazos & Nicholson, 2016) found that the mutation frequency of the *rpoB* gene, encoding the rifampicin resistance protein, in *S. epidermidis* in the spaceflight group was much higher than that in the ground group and the simulated gravity group. However, Rosado (Rosado, Stapleton, & Taylor, 2006) found that the antibiotic sensitivity of *S. aureus* grown under simulated microgravity conditions was not significantly different from that of the control group. Based on the above information, we speculate that different environments lead to different directional mutations in bacteria. The pressure of microgravity, radiation, and other factors in the space environment on bacteria can often lead to changes in physical structure and chemical damage. Therefore, if the bacteria themselves are not pathogenic or opportunistic pathogens such as *S. warneri*, changes in environmental adaptation, physical resistance, and cell repair should be evident or could even manifest as stable mutations, while changes in such aspects as drug resistance and virulence should not be significant because there is no antibiotic pressure; our results support this hypothesis.

Simultaneously, we also noticed that the control group SWG also had limited changes at both the phenotype and genetic levels compared to SWO after long‐term cultivation. Therefore, it is reasonable to assume that mutations of *S. warneri* that occur in the space environment can be inherited stably after the bacteria return to the ground. If SWO isolated directly from space can be compared with the primitive *S. warneri* on the ground to prove that the mutation is indeed caused by the influence of space environment, our assumption would be reinforced. This aspect warrants further study.

To sum up, although the changes in glycometabolism‐related transcriptome and proteome can to some extent explain the phenotypic changes such as biofilm and chemical sensitivity assay. However, the overall changes in *S. warneri* at the level of DNA or RNA are still relatively small, and there are limits to explaining the whole bacterial change in terms of changes at the RNA level.

## 5 CONCLUSION

In this study, the effects of long‐term flight on bacterial mutations were studied using *S. warneri*. The results showed that the PTS of *S. warneri* was upregulated by long‐term space stimulation, which resulted in a series of changes in the cell wall, biofilms, and chemical sensitivity, thus enhancing the resistance and adaptability of bacteria to the external environment. No other significant mutations associated with drug resistance were observed. However, as an opportunistic pathogenic bacterium, the infection and sepsis caused by *S. warneri* are diseases that cannot be easily cured even in the ground environment with complete medical care. The occurrence of similar infections in the space capsule environment would be disastrous. Therefore, in the space capsule environment, potentially pathogenic bacteria should not be neglected. The mechanism by which short‐term human‐bacterial symbiosis affects the human body and changes in the biological characteristics of staphylococci after long‐term spaceflight also warrants further exploration.

## CONFLICT OF INTERESTS

None declared.

## AUTHOR CONTRIBUTIONS

C. Liu designed and coordinated the project. P. Bai and B. Zhang performed laboratory experiments. X. Zhao, Y. Yu, X. Zhang, and D. Li performed the data analysis. P. Bai wrote the manuscript with assistance from all authors. All authors read and approved the final manuscript.

## ETHICS STATEMENT

None required.

## DATA AVAILABILITY STATEMENT

DNA and RNA data have been uploaded to the GenBank. The accession numbers of the complete genome sequences of the SWO strain are CP033098‐CP033101. Accession numbers for the resequencing data of SWG and SWS are SAMN10250545 and SAMN10255186, respectively. Accession numbers for the transcriptomic data of SWG and SWS are SAMN10255193 and SAMN10255187, respectively. The data for proteomic analysis have been uploaded to the iProX. The accession number for the proteomic data of SWG and SWS is PXD011573.
